# Senescence in Intervertebral Disc Degeneration: A Comprehensive Analysis Based on Bioinformatic Strategies

**DOI:** 10.1002/iid3.70072

**Published:** 2024-11-18

**Authors:** Zijun Zhao, Yining Wang, Zairan Wang, Fan Zhang, Ze Ding, Tao Fan

**Affiliations:** ^1^ Spine Center Sanbo Brain Hospital, Capital Medical University Beijing China; ^2^ Graduate Department Jinzhou Medical University Jinzhou China; ^3^ Department of Neurosurgery Peking Union Medical College Hospital, Chinese Academy of Medical Sciences & Peking Union Medical College Beijing China

**Keywords:** enrichment analysis, immune infiltration analysis, intervertebral disc degeneration, risk model, senescence, single cell analysis

## Abstract

**Background:**

Intervertebral disc degeneration (IDD) is a major cause for low back pain. Studies showed the association between senescence and degenerative diseases. Cell senescence can promote the occurrence and development of degenerative diseases through multiple mechanisms including inflammatory stress, oxidative stress and nutritional deprivation. The roles of senescence and senescence‐associated genes (SAGs) remains unknown in IDD.

**Methods:**

Four differently expressed SAGs were identified as hub SAGs using “limma“ package in R. We then calculated the immune infiltration of IDD patients, and investigated the relation between hub SAGs and immune infiltration. Enrichment analysis was performed to explore the functions of hub SAGs in IDD. Nomogram and LASSO model based on hub SAGs was constructed to predict the risk of severe degeneration (SD) for IDD patients. Subsequently, single cell analysis was conducted to describe the expression pattern of hub SAGs in intervertebral disc tissue.

**Results:**

We identified ASPH, CCND1, IGFBP3 and SGK1 as hub SAGs. Further analysis demonstrated that the hub SAGs might mediate the development of IDD by regulating immune infiltration and multiple pathways. The LASSO model based on the four hub SAGs showed good performance in predicting the risk of SD. Single cell analysis revealed that ASPH, CCND1 and SGK1 mainly expressed in nucleus pulposus cells, while IGFBP3 mainly expressed in epithelial cells. Eleven candidate drugs targeting hub SAGS were predicted for IDD patients through Comparative Toxicogenomics Database (CDT). PCR and immunohistochemical analysis showed that the levels of four hub SAGs were higher in SD than MD (mild degeneration) patients.

**Conclusions:**

We performed a comprehensive analysis of SAGs in IDD, which revealed their functions and expression pattern in intervertebral disc tissue. Based on hub SAGs, we established a predictive model and explored the potential drugs. These findings provide new understandings of SAG mechanism and promising therapeutic strategies for IDD.

## Graphical abstract

Our research performed a comprehensive analysis of senescence‐associated genes (SAGs) in intervertebral disc degeneration, which revealed their functions and expression pattern in intervertebral disc tissue. We identified four hub SAGs, and established a predictive nomogram and explored the potential drugs. Our reserch provide new understandings of SAG mechanism and promising therapeutic strategies for IDD patients.

## 1 Introduction

Intervertebral disc degeneration (IDD) is considered as the main contributor for low back pain, often caused by the function and structure disorder of disc and its surrounding area. Worldwide, approximately 90% of people over 55 years old and 40% of people under 30 years old suffer from some form IDD in varying degrees [1, 2]. Currently, treatment modalities for IDD encompass rest, analgesics and surgical intervention [3]. Nevertheless, these established approaches merely provide symptomatic relief and do not impede disease progression [4, 5]. Investigating strategies to postpone the onset and advancement of IDD has emerged as a prominent area of research. IDD is initiated by intricate cellular apoptosis and senescence mechanisms. Senescent cells and the senescence‐associated secretory phenotype (SASP) can excessively produce inflammation and metabolic factors that hinder normal tissue homeostasis, culminating in a disruption of intervertebral disc matrix homeostasis [6, 7]. The upregulation of ECM proteases, specifically matrix metalloproteinases (MMPs), contributes to ECM degradation, a critical process in the reduction of structural integrity in the intervertebral disc (IVD). The phenotypic changes observed in senescent IVD cells disrupt the equilibrium between extracellular matrix (ECM) synthesis and breakdown, thereby hastening the progression of IDD [8, 9].

Cellular senescence is typically characterized by the process of permanent proliferation arrest of cells and physiological dysfunction of cells during life activities, driven by sustained exogenous and endogenous stress and damage [8, 10, 11]. Senescent cells, as products of the cellular senescence process, have special biological characteristics and functions, and are one of the important reasons for damaged tissue regeneration and chronic age‐related diseases. The mechanisms contributing to cellular senescence in intervertebral disc (IVD) encompass telomere shortening, DNA damage, oxidative stress, and dysregulation of pro‐inflammatory factors [12]. NOX4 mediates the generation of reactive oxygen species (ROS) via the p53 p21‐Rb and p16‐Rb pathways, leading to DNA damage and activation of MAPK and NF‐κB, resulting in cell cycle arrest, matrix degradation metabolism, and pro‐inflammatory gene expression in nucleus pulposus (NP) cells [13, 14].

Senescent IVD cells exhibit a reduced capacity for cell proliferation, resulting in a gradual decline in the number of functional cells within the intervertebral disc [15]. These cells experience a diminished ability to synthesize new matrix proteoglycans and an increased degradation of existing matrix proteoglycans, ultimately contributing to the development of IDD [16, 17]. Furthermore, senescent IVD cells have been found to exhibit SASP, which exerts a pronounced catabolic effect on neighboring cells and ECM, leading to tissue and functional impairment [18]. The release of pro‐inflammatory cytokines from senescent IVD cells, such as IL‐1, IL‐6, IL‐8, and TNF‐α has been shown to contribute to the aging of neighboring IVD cells and the infiltration of immune cells, leading to increased inflammation and oxidative stress within the microenvironment of degenerative IVD [17, 19]. In IDD patients, notable elevations were noted in the levels of monocytes/macrophages, eosinophils (Eos), and basophils. Among these immune cell types, macrophages play a pivotal role in the occurrence and progression of IDD [20]. M1 macrophages release pro‐inflammatory cytokines such as IL‐1 and TNF‐α, contributing to an inflammatory microenvironment and hastening ECM degradation. The inflammatory microenvironment fosters the polarization of macrophages towards the M1 phenotype, resulting in increased secretion of pro‐inflammatory cytokines. This detrimental cycle hastens the senescence process of NP cells, ultimately resulting in intervertebral disc degeneration [21].

**Figure 1:**
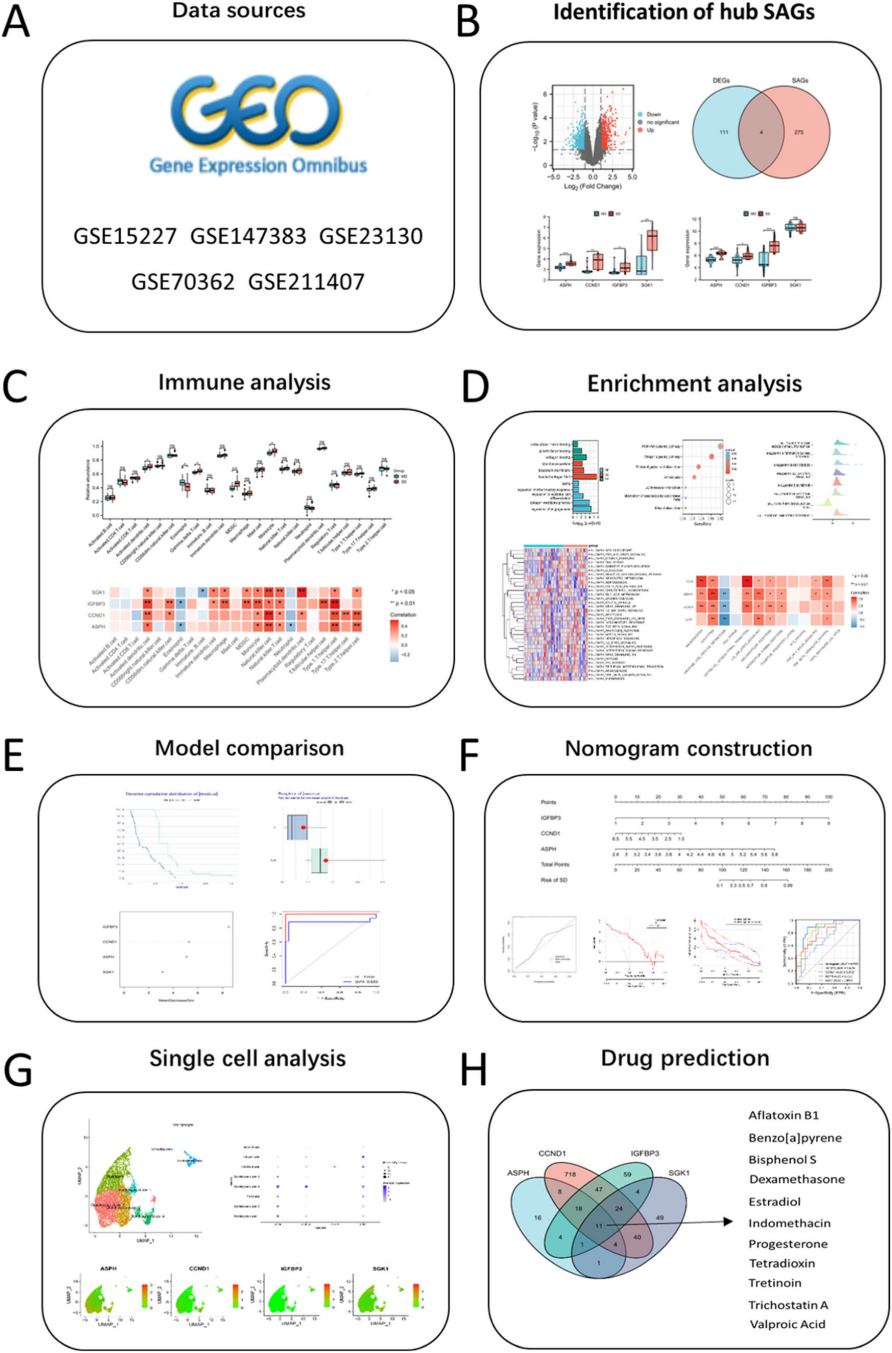
The flowchart of the study. (A) Data acquisition from GEO database. (B) Identification of hub SAGs in IDD using DEG analysis and Venn Diagram. (C) Investigation of the relation between hub SAGs and immune infiltration using ssGSEA. (D) Enrichment analysis was conducted to explore the association between hub SAGs and various cellular pathways. (E) Comparison of SVM and RF model to identify the candidate SAGs for nomogram establishment. (F) A nomogram was developed to better predict the risk of SD for IDD patients. (G) Single cell analysis reflected the expression pattern of hub SAGs in IDD tissue. (H) Prediction of potential drugs targeting SAGs for IDD patients.

Previous studies have shown that SAG may play an important role in the development of degenerative diseases. In osteoarthritis (OA), Geng et al. found that overexpression of MAPK12 and knockout of FOS can promote cell proliferation and cartilage synthesis metabolism, inhibit cell aging and cartilage degradation metabolism, providing potential therapeutic targets for the prevention or treatment of OA in the future [22]. Guo et al. found that STING promotes OA by activating the NF‐κB signaling cascade, and knocking down STING alleviated the instability of OA development induced by the medial meniscus in mice [23]. In Parkinson's disease (PD), SATB1 has recently been identified as a risk factor for PD, with reduced activity observed in the brain regions most susceptible to PD. SATB1 protects dopaminergic neurons from aging in vivo [24]. While there exist numerous descriptive studies on the senescence process of intervertebral disc cells, further research is necessary to elucidate the mechanisms by which senescence‐related genes contribute to IDD in the context of senescence [25, 26]. Additionally, it is imperative to investigate the relationship between these genes and various cellular pathways, including apoptosis, autophagy and inflammation, as well as the immune microenvironment [11, 27]. This is essential for comprehending the impact of senescence on IDD and developing strategies to prevent, delay, or ameliorate the progression of age‐related IDD. Herein, we identified hub senescence‐related genes (SAGs) using bioinformatics analysis. The specific pathways and biological functions of SAGs were analyzed utilizing gene set enrichment analysis (GSEA) and gene set variation analysis (GSVA) analysis. Immune infiltration analysis was performed to investigate the association between hub SAGs and immune microenvironment in IDD. A nomogram model was established based on the hub SAGs, which could predict the risk of severe degeneration (SD). Finally, potential drugs targeting hub SAGS were predicted for IDD patients through CDT database (Figure 1). Our study provides new understandings of SAG mechanism in IDD, as well as potential therapies for IDD patients.

## 2 Materials and Methods

### 2.1 Data Acquisition

Four IDD datasets (GSE15227: 15 samples [28], GSE147383: 8 samples [29], GSE23130: 23 samples [30], GSE70362: 24 samples [31]) including RNA sequencing data were downloaded from Gene Expression Omnibus (GEO, http://www.ncbi.nlm.nih.gov/geo/) database. Mild degeneration (MD) means samples from Grade I to Grade III. SD samples of Grade IV and Grade V. GSE15227 data set includes 3 degenerative discs tissues and 12 healthy disc tissues. GSE147383 includes four IDD samples (two nucleus pulposus tissues and two annulus fibrosus tissues) and four healthy controls (two nucleus pulposus tissues and two annulus fibrosus tissues). GSE23130 includes 23 intervertebral disc tissues (MD: 8 cases, SD: 15 cases). GSE70362 includes 23 intervertebral disc tissues (MD: 14 cases, SD: 10 cases). The sample information of the four datasets is showed in Table 1.

**Table 1:** Data set information.

Data set	Research Population	Control Population	Tissue resource
GSE15227	3 (IDD)	12 (healthy control)	Disc tissues
GSE147383	4 (IDD)	4 (healthy control)	Disc tissues
GSE23130	8 (MD)	15 (SD)	Disc tissues
GSE70362	14 (MD)	10 (SD)	Disc tissues

### 2.2 Identification of hub SAGs in IDD

The senescence‐associated genes (SAGs) were acquired from CellAge platform (https://genomics.senescence.info/cells/). The “limma“ package in R was performed to investigate differently expressed genes (DEGs) between IDD and healthy samples in GSE15227 and GSE147383. The screening criteria were *p* < 0.05 and |log2 fold‐change (FC) | > 1. The ggplot2 package in R was used to plot the volcano plot for the DEGs. The overlap of DEGs (GSE15227), DEGs (GSE147383) and SAGs were considered as hub SAGs. Then the expression of hub SAGs was validated in MD and SD samples in GSE23130 and GSE70362.

### 2.3 Immune Infiltration Estimation

To estimate the relative abundance of 23 immune cell types, we used the R package “GSVA” to conduct ssGSEA (single sample gene set enrichment analysis) [32]. The gene set signatures of the 23 immune cell types were obtained from a previous study [33]. Herein, we explored the relative abundance of 23 immune cell types in MD and SD population, and then analyzed the association between the 4 hub SAGs and 23 immune cell types.

### 2.4 Identification of DEGs Between MD and SD Population

The “limma“ package in R was performed to explore the DEGs between MD and SD population in the combined data set (screening criteria: *p* < 0.05 and |log2 fold‐change (FC) | > 0.5). The protein‐protein interaction (PPI) network of DEGs was drawn utilizing STRING platform (http://string-db.org/) and Cytoscape software. We then analyzed the DEGs in the Kyoko Encyclopaedia of Genes and Genomes (KEGG) and Gene Ontology (GO) database using the “clusterProfiler” package in R software [34]. GSEA analysis was subsequently performed to explore the potential pathways between SD and MD population.

GSVA is an unsupervised and non‐parametric method for the estimation of certain biological process and pathway based on the sequence data [32]. Herein we calculated the GSVA scores of multiple pathways and biological processes in the combined data set. To investigate the relation between hub SAGs and potential pathways of IDD, we performed correlation analysis between SAG expression and the GSVA scores.

### 2.5 Establishment of Support Vector Machine (SVM) and Random Forest (RF) Model for Candidate Sags

SVM and RF are two widely utilized machine learning algorithms. To predict the risk of SD, SVM and RF model were established using “randomForest” and “kernlab” package in R. We subsequently drew receiver operating characteristic (ROC) curve, “Boxplots of residual” and “Reverse cumulative distribution of residual” to measure the performance of our models. After comparing the two models, we selected the better one to identify candidate SAGs.

### 2.6 Nomogram Establishment

Based on the candidate SAGs, a nomogram was developed using “RMS” package in R to forecast the risk of SD. To explore the consistency between the predicted and actual values, we drew the calibration curves. Clinical impact curve and decision curve were also plotted to evaluate predictive values of the nomogram.

### 2.7 Least Absolute Shrinkage and Selection Operator (LASSO) Regression Analysis

To predict the risk of SD in IDD patients, we constructed a LASSO model utilizing the gene expression profiles of hub SAGs via the “glmnet” package in R. The optimal variables in the LASSO model were selected based on the minimum λ value. Following this, regression analysis was conducted on the genes identified by the LASSO model, and regression coefficients for the candidate genes were computed using the designated formula.

### 2.8 Single Cell Sequencing Analysis

The data of degenerative intervertebral disc for single cell analysis were downloaded from GSE211407 (https://www.ncbi.nlm.nih.gov/geo/query/acc.cgi?acc=GSE211407). The “Seurat” package in R was performed for data preprocessing and cell clustering, and CellMarker (http://bio-bigdata.hrbmu.edu.cn/CellMarker/) database was used to identify the different cell types.

### 2.9 Prediction of Potential Drugs Targeting the Four Hub SAGs

The Comparative Toxicogenomics Database (http://ctdbase.org/, CTD), is a publicly available database for chemical‐gene, chemical‐phenotype and chemical‐disease association [35]. We utilized CTD database to identify the potential drugs targeting SAGs for IDD patients.

### 2.10 Tissue Samples

Human nucleus pulposus (NP) tissues of IDD were acquired from 31 individuals undergoing surgery at Beijing Sanbo Brain Hospital between 2022 and 2024 (MD: 13 cases, SD: 18 cases). Written informed consent was obtained from all subjects before the study. Based on Helsinki Declaration, the Ethics Committee of Sanbo Brain Hospital approved this study (SBNK‐YJYS‐2024‐030‐01).

### 2.11 RNA Extraction and Reverse Transcription‐Polymerase Chain Reaction (RT‐PCR)

The TRIzol method was used to extract total RNA. Reverse transcriptase was used to synthesize complementary DNA. We measured mRNA levels of the target gene by RT‐PCR amplification using GAPDH as the internal control. The primers used for quantification of relative mRNA expression were as follows: GAPDH (5′‐TGACTTCAACAGCGACACCCA‐3′ and 5′‐CACCCTGTTGCTGTAGCCAAA‐3′), ASPH (5′‐GTTACCACGTGGAAGAGAC‐3′ and 5′‐GCTTGTTCCTCATAGACTTG‐3′), CCND1(5′‐GGAGCCTATTCTGCCCATTT‐3′ and 5′‐CGAGGTCATAGTTCCTGTTGGTG‐3′), IGFBP3 (5′‐AGAGCACAGATACCCAGAACT‐3′ and 5′‐GGTGATTCAGTGTGTCTTCCA‐3′), SGK1 (5′‐TTCTGTGGCACGCCTGAGTAand GGTCGTACGGCTGCTTATGG).

### 2.12 Immunohistochemical Analysis

Paraffin‐embedded sections from 31 patients, comprising 13 samples from the MD group and 18 samples from the SD group, underwent antigen retrieval using 1% sodium citrate (Bioss, Beijing, China). These sections were subsequently incubated overnight at 4℃ with antibodies targeting ASPH (1:100 dilution, ab172475, Abcam), CCND1 (1/200 dilution, ab16663, Abcam), IGFBP3 (1/200 dilution, ab217205, Abcam), and SGK1 (1:100 dilution, ab32374, Abcam). Following this, the sections were treated with horseradish peroxidase (HRP)‐conjugated secondary antibodies at room temperature for 1 h. Nuclear counterstaining was performed using hematoxylin. Microscopic imaging (Olympus) was then conducted to capture the sections.

## 3 Results

### 3.1 Identification and Validation of Hub SAGs

**Figure 2:**
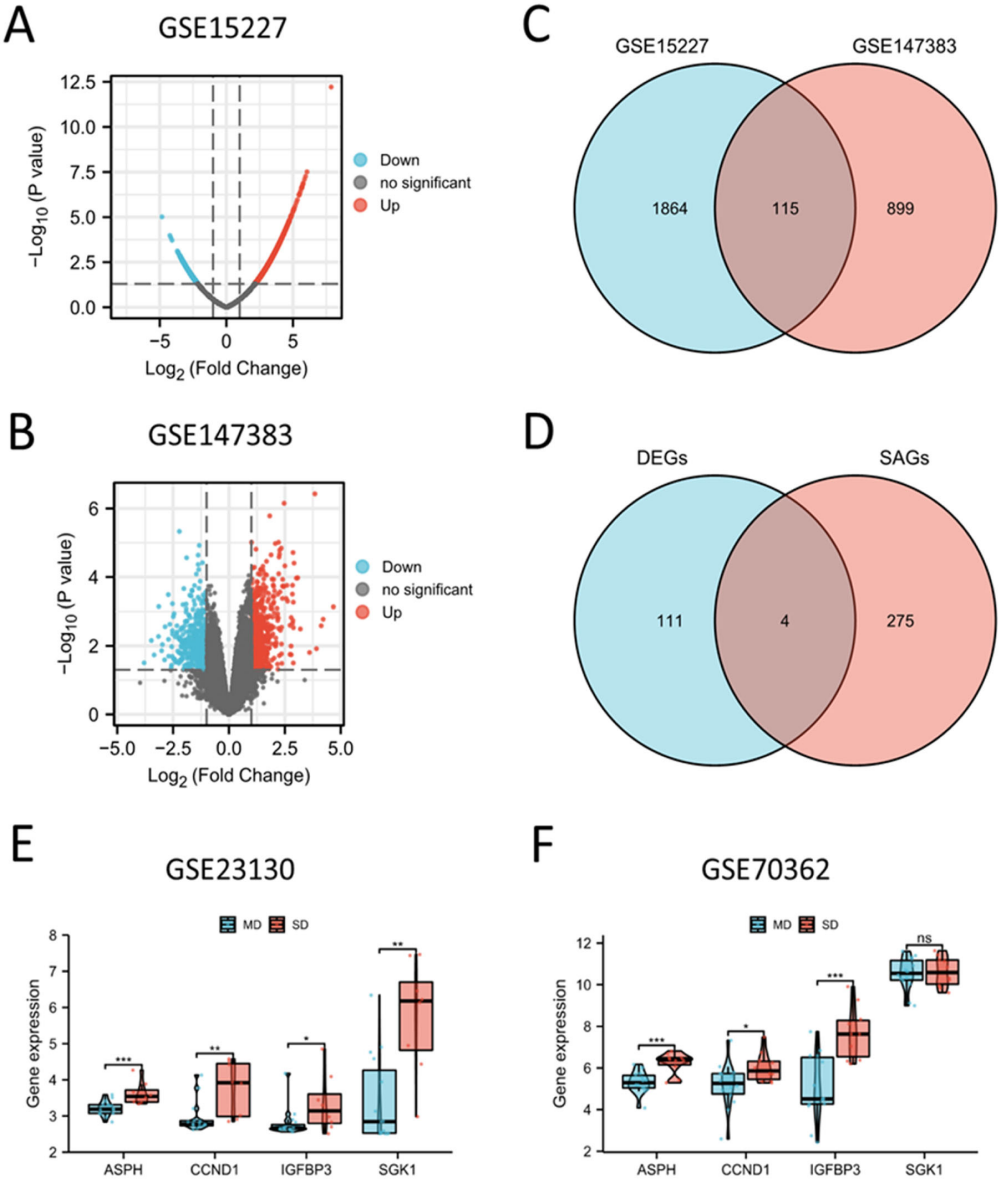
Identification and validation of hub SAGs. (A) The DEGs between IDD and healthy control in GSE15227. (B) The DEGs between IDD and healthy control in GSE147383. (C) The overlap of the DEGs in GSE15227 and GSE147383 had 115 genes. (D) The overlap of 115 DEGs and 279 SAGs. (E, F) Validation of the four hub SAGs in GSE23130 and GSE70362. ns, no significance; *p < 0.05; **p < 0.01; ***p < 0.001.

By analyzing the DEGs between IDD tissues and healthy controls, a total of 1979 DEGs were acquired from GSE15227 (Figure 2A), and 1,014 DEGs were acquired from GSE147383 (Figure 2B). The screening criteria were *p* < 0.05 and |log2FC | > 1. A Venn diagram identified 115 overlapping genes between GSE15227 and GSE147383 (Figure 2C and Table S1). By taking the intersection of the 115 genes and 279 SAGs, we obtained 4 hub SAGs: ASPH, CCND1, IGFBP3 and SGK1 (Figure 2D). Then we validated the specific expression of hub SAGs in GSE23130 and GSE70362. Our results illustrated that IDD patients in SD population had higher expression of four hub SAGs than patients in MD population (Figure 2E,F).

**Figure 3:**
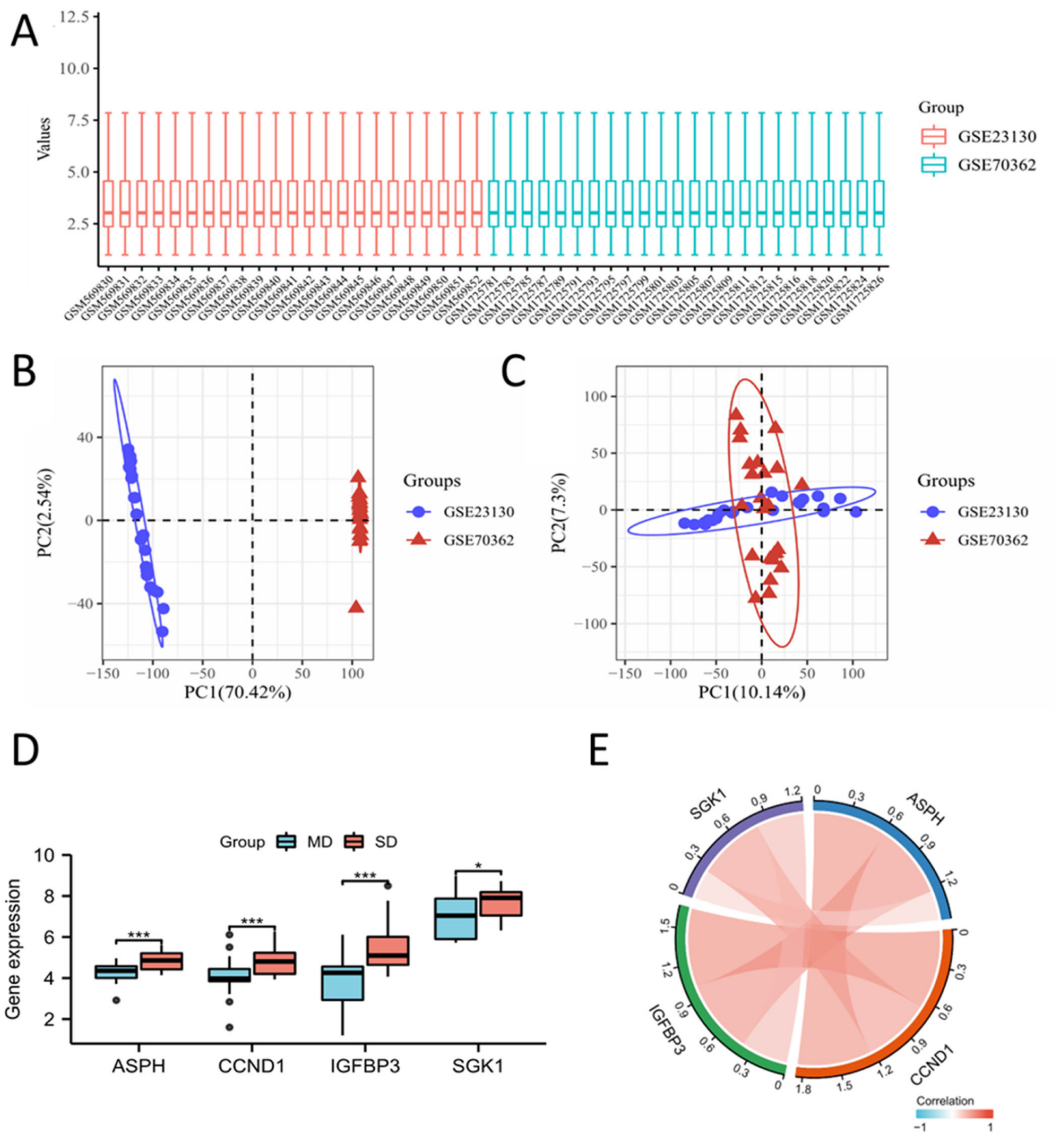
Combination of GSE23130 and GSE70362. (A) The boxplot of the normalized data. Different colors represent different datasets. Rows represent samples, and columns represent the gene expression values in the samples. (B) PCA results before batch removal for multiple datasets. Different colors represent different datasets. As shown in the schematic diagram, the two datasets are separated without any intersection. (C) PCA results after batch removal, as shown in the schematic diagram shows the intersection of two datasets, which can be used in subsequent analysis. (D) Expression of four hub SAGs in the combined data set. (E) The hub SAGs positively correlated with each other in the combined data set. ns, no significance; *p < 0.05; **p < 0.01; ***p < 0.001.

To provide a better analysis of the sequencing data, we combined GSE23130 and GSE70362 and normalized their microarray data (Figure 3A). PCA analysis was conducted to describe sample correlation (Figure 3B,C). We subsequently investigated the expression of four hub SAGs in the combined data set (Figure 3D), and found that hub SAGs positively correlated with each other (Figure 3E).

### 3.2 Analysis of Immune Infiltration

**Figure 4:**
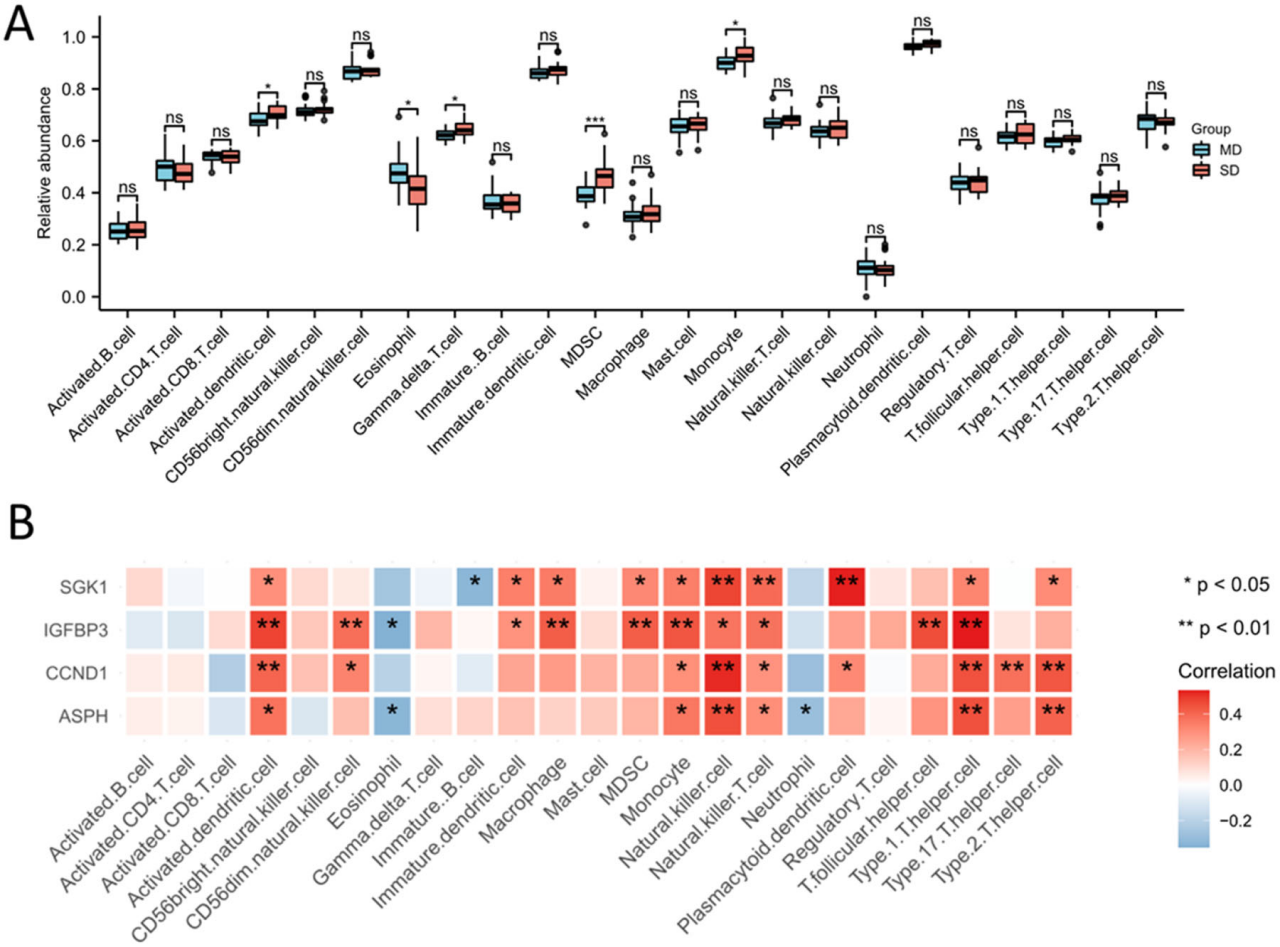
Immune infiltration of IDD. (A) The relative abundance of 23 immune cell types in MD and SD population. (B) Correlation analysis of the four hub SAGs and 23 immune cell types. ns, no significance; *p < 0.05; **p < 0.01; ***p < 0.001.

The ssGSEA analysis was performed to estimate the relative abundance of 23 immune cell types in the combined data set. The results demonstrated that gamma delta T cells, MDSCs (marrow‐derived suppressor cells) and monocytes were higher in SD population than that in MD population, and eosinophils were higher in MD population than that in SD population (Figure 4A). The correlation analysis showed that the hub SAGs were positively correlated with activated dendritic cells, MDSCs, monocytes, natural killer cells and type 1 T‐helper cells (Figure 4B).

### 3.3 Relation Between Hub SAGs and Multiple Pathways in IDD

**Figure 5:**
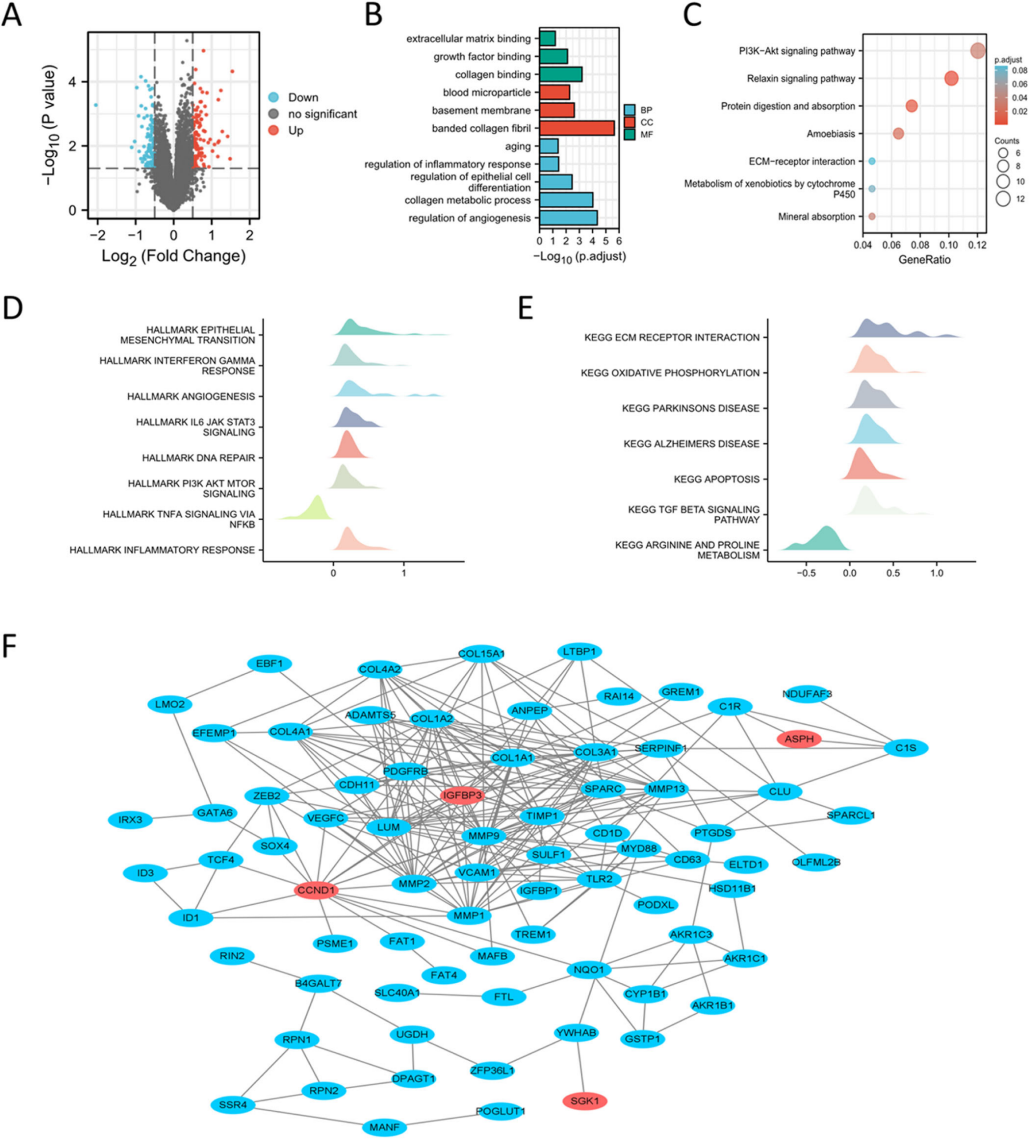
Enrichment analysis and PPI network. (A) DEGs between SD and MD population in the combined data set. (B) GO analysis of DEGs. (C) KEGG analysis of DEGs. (D‐E) GSEA analysis displayed the pathways and biological processes enriched in SD population. (F) PPI network of DEGs. Four hub SAGs were highlighted in red.

111 DEGs between SD and MD population were obtained and visualized (Figure 5A and Table S2). GO and KEGG analysis demonstrated that DEGs were enriched in aging, regulation of inflammatory response, regulation of epithelial cell differentiation and regulation of angiogenesis and PI3K‐Akt signaling pathway (Figure 5B,C). GSEA analysis showed that epithelial mesenchymal transition, interferon gamma response, IL6‐JAK‐STAT3 pathway, inflammatory response and apoptosis were enriched in SD population (Figure 5D,E). PPI network exhibited the association between hub SAGs and other DEGs (Figure 5F). CytoHubba algorithm was then used to estimate the importance of DEGs, and the results were showed in Table S3. The CytoHubba results for the four hub SAGs were showed in Table 2.

**Table 2:** The CytoHubba results for the four hub SAGs.

Gene name	Degree	MCC	BottleNeck	Closeness	Stress	Betweenness
IGFBP3	12	12,264	1	36.04802	714	81.13251
CCND1	16	1699	13	39.29802	5976	1580.77767
ASPH	2	2	1	21.59845	0	0
SGK1	1	1	1	18.1131	0	0

**Figure 6:**
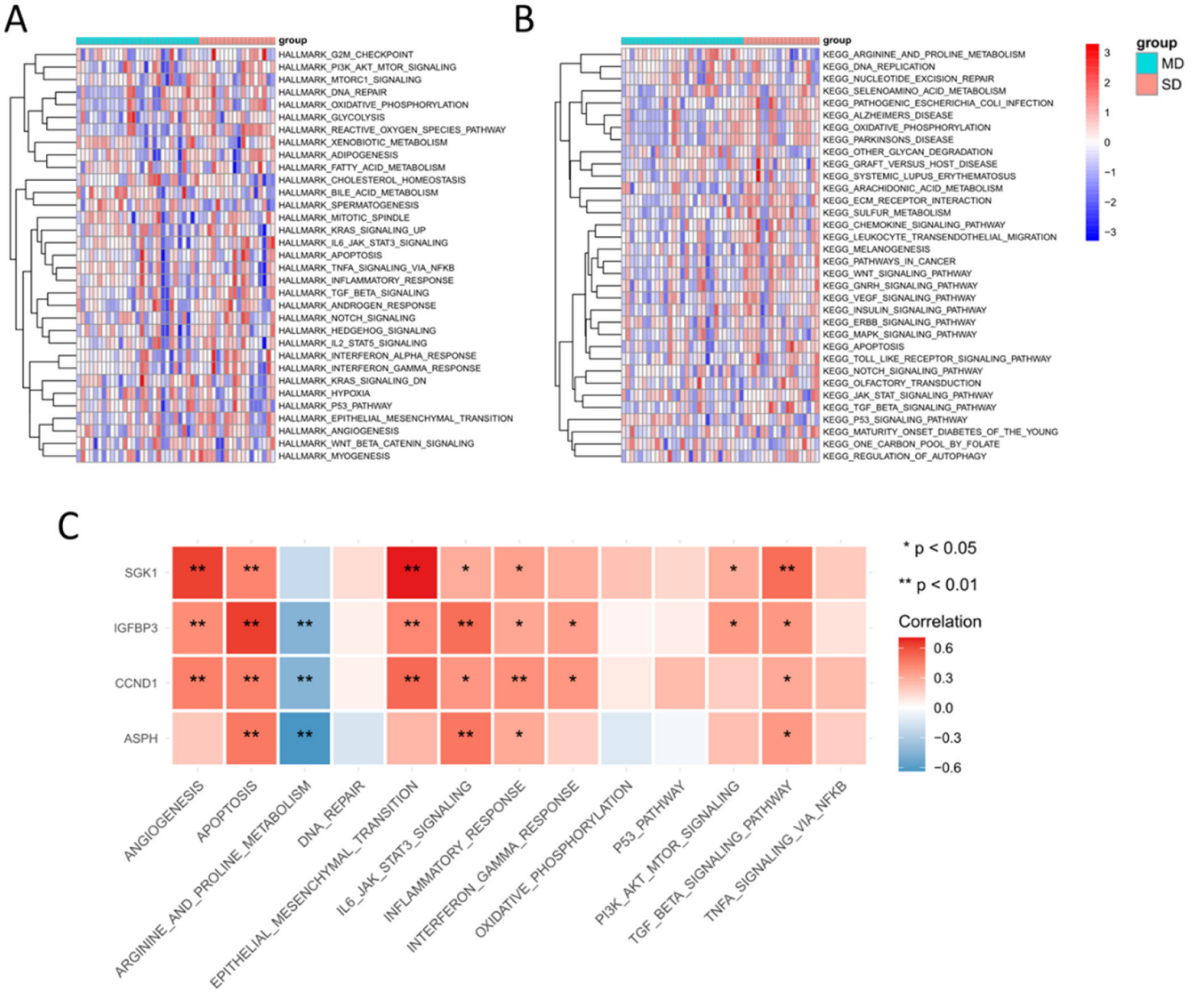
GSVA analysis. (A, B) Heatmap showed the GSVA scores of multiple pathways and biological processes in the combined data set. (C) Association between hub ARGs and various cellular pathways. *p < 0.05; **p < 0.01.

Then we performed GSVA analysis to calculate the enrichment scores of Hallmark and KEGG gene sets. The results showed that PI3K‐Akt signaling pathway, IL6‐JAK‐STAT3 pathway, TGF‐β signaling pathway, epithelial mesenchymal transition, DNA repair, glycolysis and apoptosis were relatively activated in SD population (Figure 6A,B). In addition, the association between hub SAGs and these pathways was investigated (Figure 6C).

### 3.4 Establishment of SVM and RF Model

**Figure 7:**
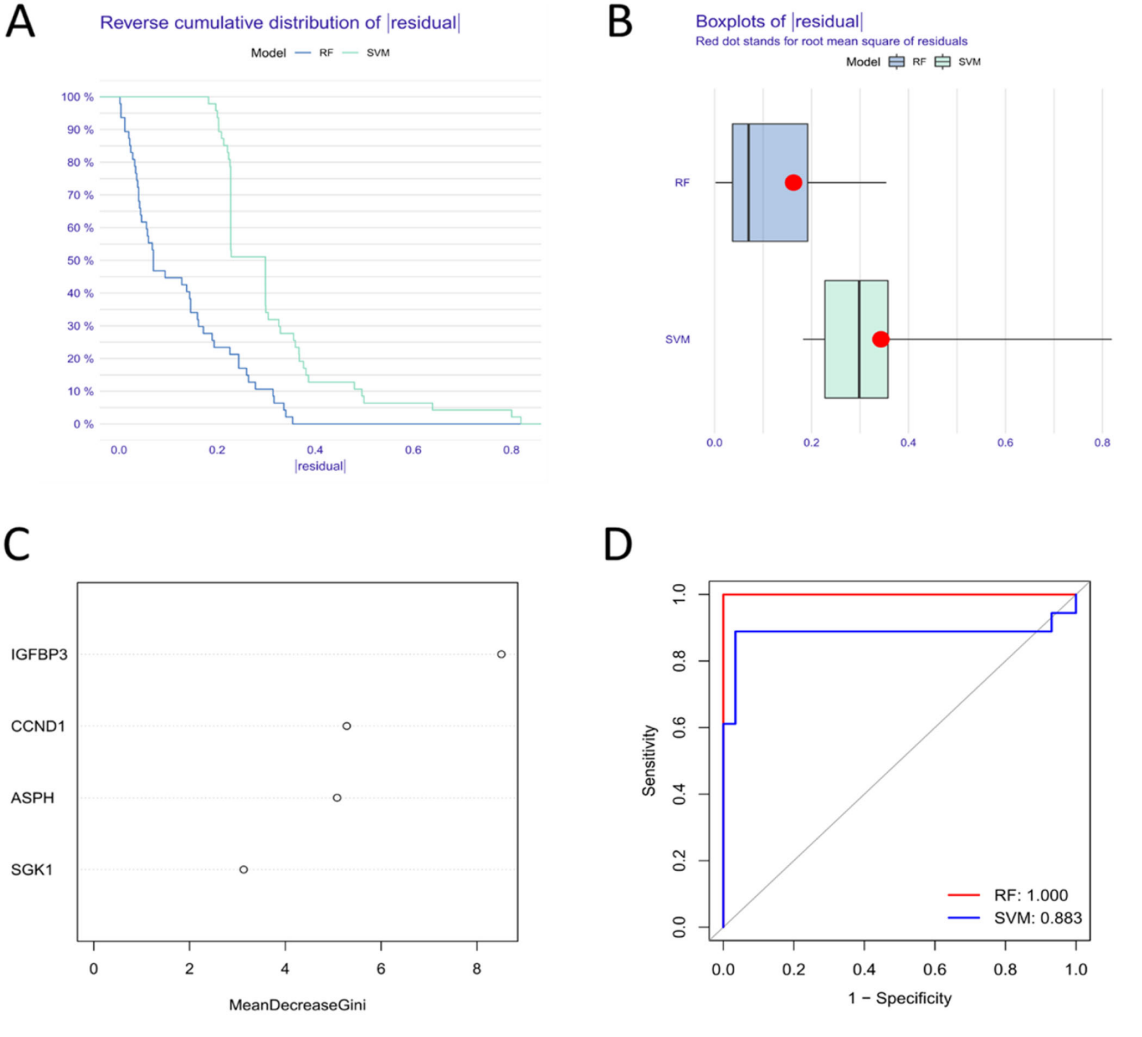
Identification of candidate SAGs using machine learning algorithms. (A) The residual distribution of SVM and RF model plotted in reverse cumulative distribution of residual. (B) The residual distribution of SVM and RF model plotted in boxplot. (C) The hub SAGs were visualized after ranking genes based on importance, and three SAGs (IGFBP3, CCND1 and ASPH) with importance scores greater than four. (D) ROC curve analysis revealed that the RF model (AUC = 1.000) was more accurate than the SVM model (AUC = 0.883).

SVM and RF model were developed based on the four hub SAGs to predict the risk of SD. The results of “Reverse cumulative distribution of residual” (Figure 7A), “Boxplots of residual” (Figure 7B) and ROC curve (Figure 7D) all showed that RF model has better predictive values than SVM model. We identified hub SAGs following the ranking of genes according to their importance scores. Three SAGs (IGFBP3, CCND1 and ASPH) exhibited importance scores higher than 4 (Figure 7C). Considering the low importance of SGK1, we selected IGFBP3, CCND1 and ASPH as candidate SAGs for further analysis.

### 3.5 Nomogram Construction

**Figure 8:**
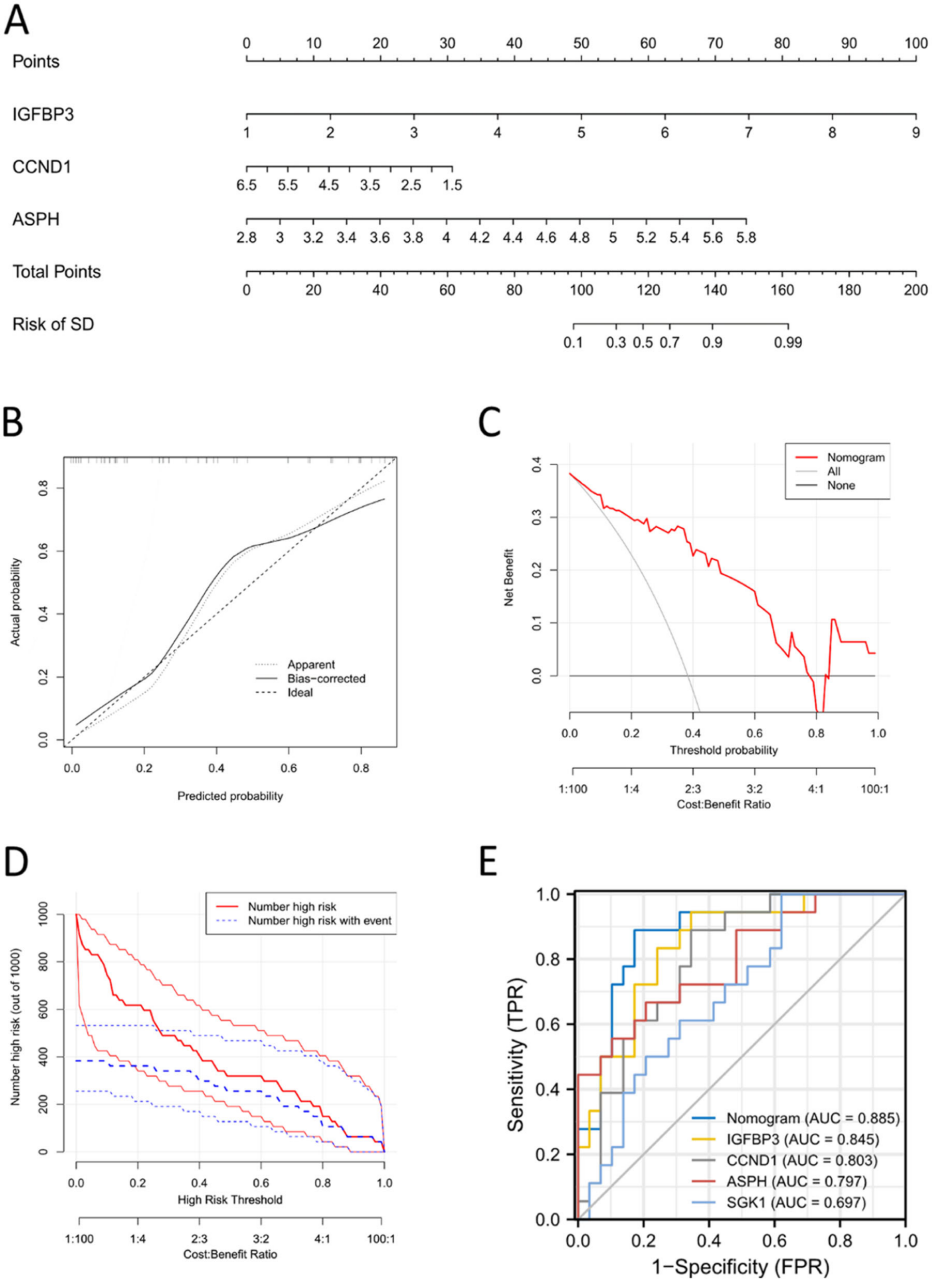
Establishment of a nomogram to predict the risk of severe degeneration in IDD. (A) Nomogram development based on three SAGs: IGFBP3, CCND1 and ASPH. (B) Based on the results of the calibration curve, we confirm that the nomogram predictions are accurate. (C) Based on the DCA curve, patients with IDD may benefit from decision‐making based on the nomogram, since the red lines are consistently maintained above the gray and black lines of 0–1. (D) Clinical impact curve showed the significant predictive ability for the nomogram model. (E) ROC curve showed prediction ability of the nomogram compared to other indexes.

A nomogram was constructed using the three candidate SAGs (IGFBP3, CCND1 and ASPH) (Figure 8A). The calibration curve of the nomogram was consistent with the standard curve (Figure 8B). The decision curve (Figure 8C) and clinical impact curve (Figure 8D) both revealed the good performance of our nomogram in predicting the risk of SD. The ROC curve showed that the nomogram had a better predictive value than the four SAGs (Figure 8E).

### 3.6 Construction and the Validation of LASSO Model

To better predict the risk of SD for IDD patients, we analyzed the expression profiles of IGFBP3, CCND1, and ASPH, subsequently developing a LASSO model (Figure S1A,B). Ultimately, IGFBP3 and ASPH were selected to formulate the model index, or risk score, using the following equation: risk score = (0.797) × IGFBP3 + (1.587) × ASPH − 11.496. The model's accuracy was evaluated using receiver operating characteristic (ROC) curves and quantified by the area under the curve (AUC) values in training data set (Figure S1C; ASPH: 0.797, CCND1: 0.803, IGFBP3: 0.845, SGK1: 0.697, Risk score: 0.887). We then validated the model's predictive accuracy with a cohort of 31 samples from our hospital (validation data set), demonstrating its robust predictive capability as indicated by the AUC values (Figure S1D; ASPH: 0.880, CCND1: 0.848, IGFBP3: 0.904, SGK1: 0.763, Risk score: 0.927).

### 3.7 Single Cell Analysis Reflected the Expression Pattern of Four Hub SAGs in IDD Tissue

**Figure 9:**
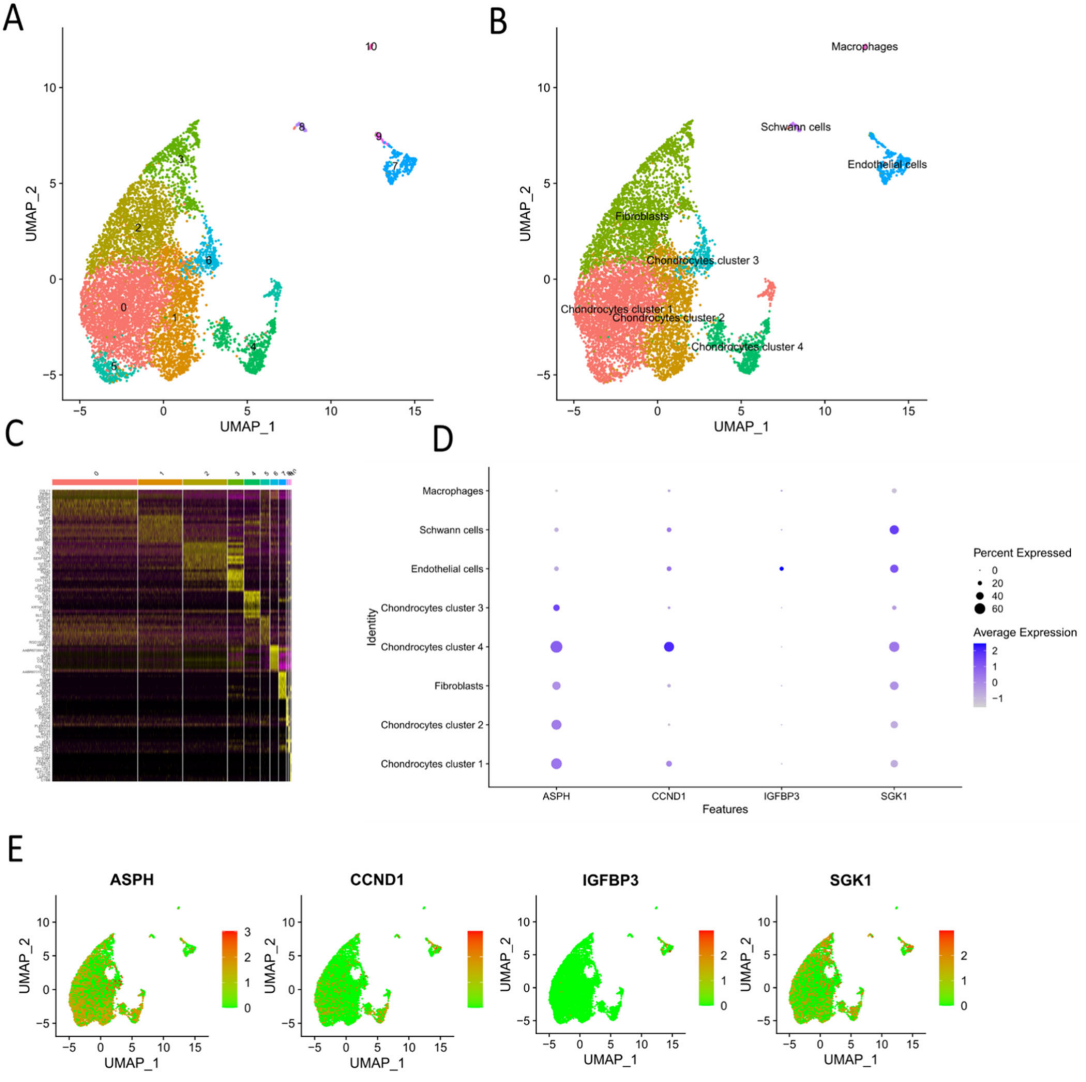
Single cell analysis revealed the expression patterns of the four hub genes in degenerated IVD. (A) T‐SNE analysis identified 11 cell clusters in IVD tissues. (B) Cell clusters were identified based on cell‐specific markers. (C) The top 10 marker genes of the 11 cell clusters. (D, E) Expression pattern of four hub SAGs in different cell clusters.

Using “Seurat” package, we acquired 11 cell clusters (Figure 9A) and the marker genes for each cell cluster (Figure 9C). Finally, five cell types (chondrocytes, fibroblasts, Schwann cells, endothelial cells and macrophages) were identified utilizing specific marker genes in CellMarker database (Figure 9B). The chondrocytes could be divided into 4 clusters: chondrocytes cluster 1–4. Then, the expression pattern of four hub SAGs were investigated in these cell clusters. The results showed that ASPH was mainly expressed in chondrocytes (nucleus pulposus cells) and fibroblasts (annulus fibrosus cells); CCND1 was mainly expressed in chondrocytes (nucleus pulposus cells) cluster 4; IGFBP3 was mainly expressed in endothelial cells; SGK1 was expressed in almost all cell types (Figure 9D,E).

**Figure 10:**
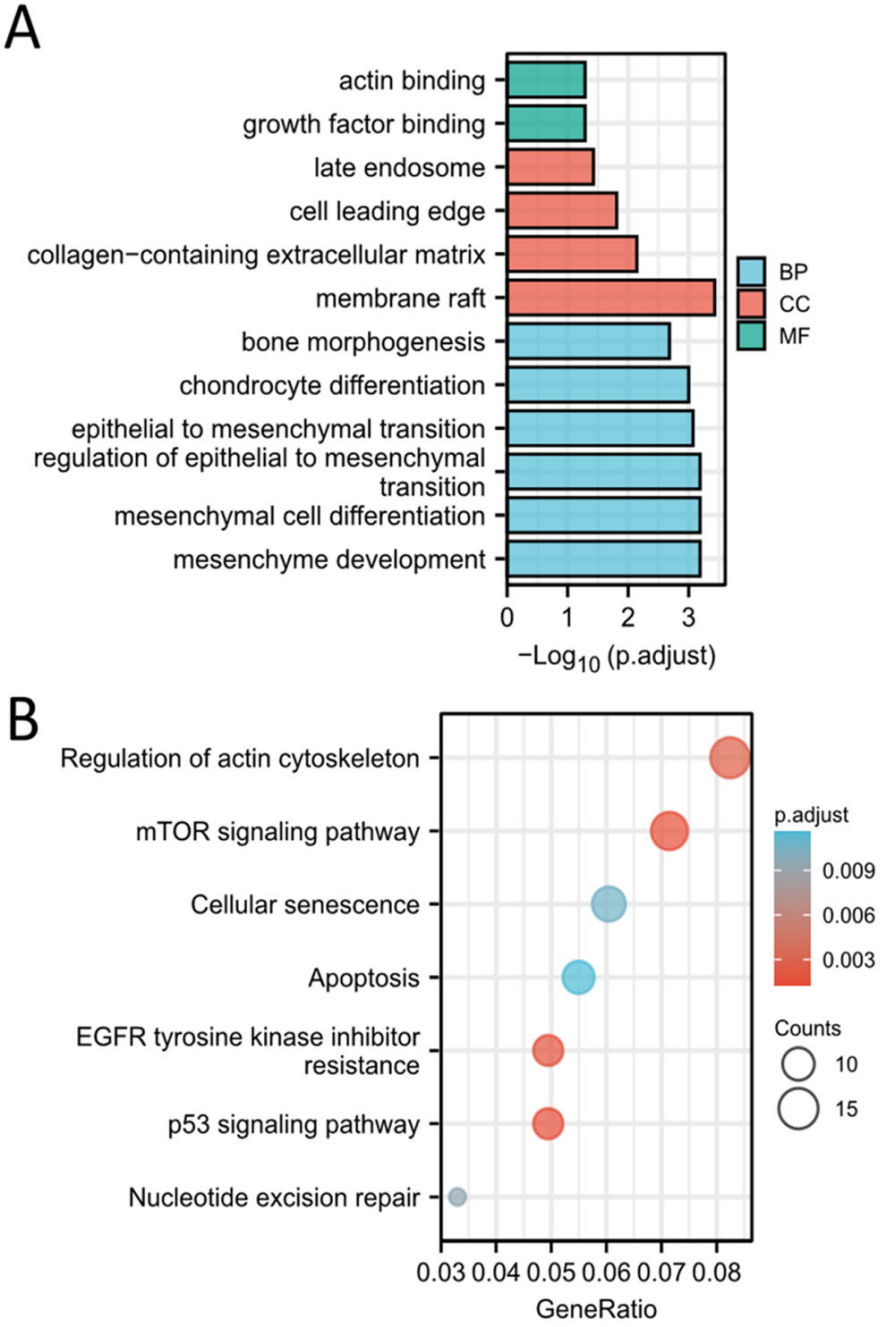
Functional analysis of chondrocytes cluster 4. (A) GO analysis of the marker genes for chondrocytes cluster 4. (B) KEGG analysis of the marker genes for chondrocytes cluster 4.

To exactly know the functions of chondrocytes cluster 4, we conducted the GO and KEGG enrichment analysis based on the specific marker genes of chondrocytes cluster 4. This list of the marker genes was showed in Table S1. The results demonstrated that the marker genes were enriched in chondrocyte differentiation, epithelial to mesenchymal transition, mesenchymal cell differentiation and nucleotide excision repair (Figure 10A,B). Therefore, we thought that chondrocytes cluster 4 were the “repair cells” in nucleus pulposus.

### 3.8 Prediction of Potential Drugs Targeting Sags for IDD Patients

**Figure 11:**
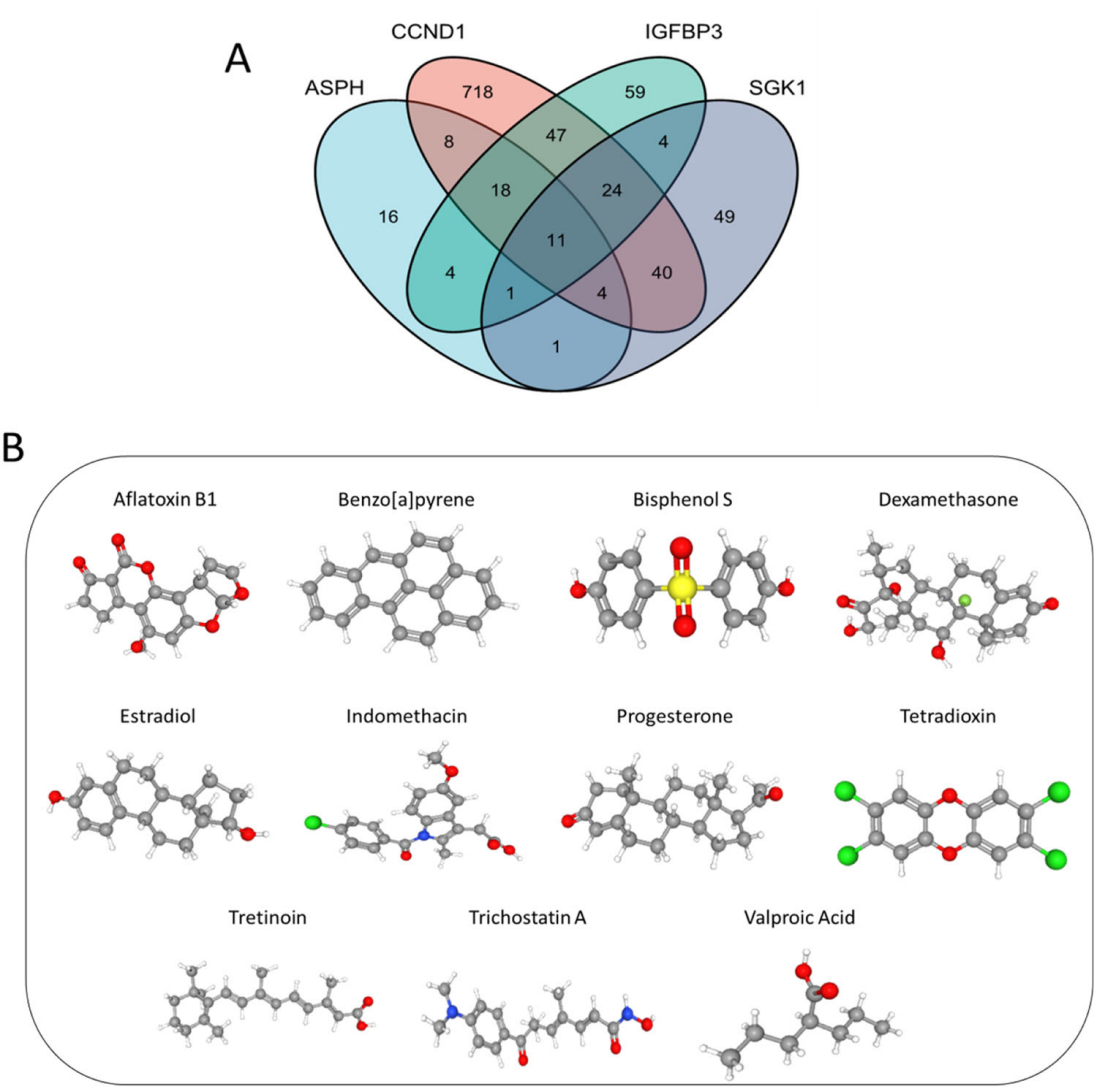
Prediction of potential drugs. (A) Identification of 11 chemicals targeting the 4 hub SAGs using Venn diagram. (B) The 3D structure of the 11 chemicals from PubChem database.

To identify chemicals targeting the 4 hub SAGs, we searched their gene names in CDT database, and obtained 63 chemicals for ASPH, 870 chemicals for CCDN1, 168 chemicals for IGFBP3 and 134 chemicals for SGK1. After taking the intersection, we finally obtained 11 chemicals targeting the 4 SAGs (Figure 11A). The 3D structure of the 11 chemicals was acquired from PubChem (https://pubchem.ncbi.nlm.nih.gov/) database (Figure 11B).

### 3.9 Hub SAGs Expression Validation Using Immunohistochemical and PCR Analysis

**Figure 12:**
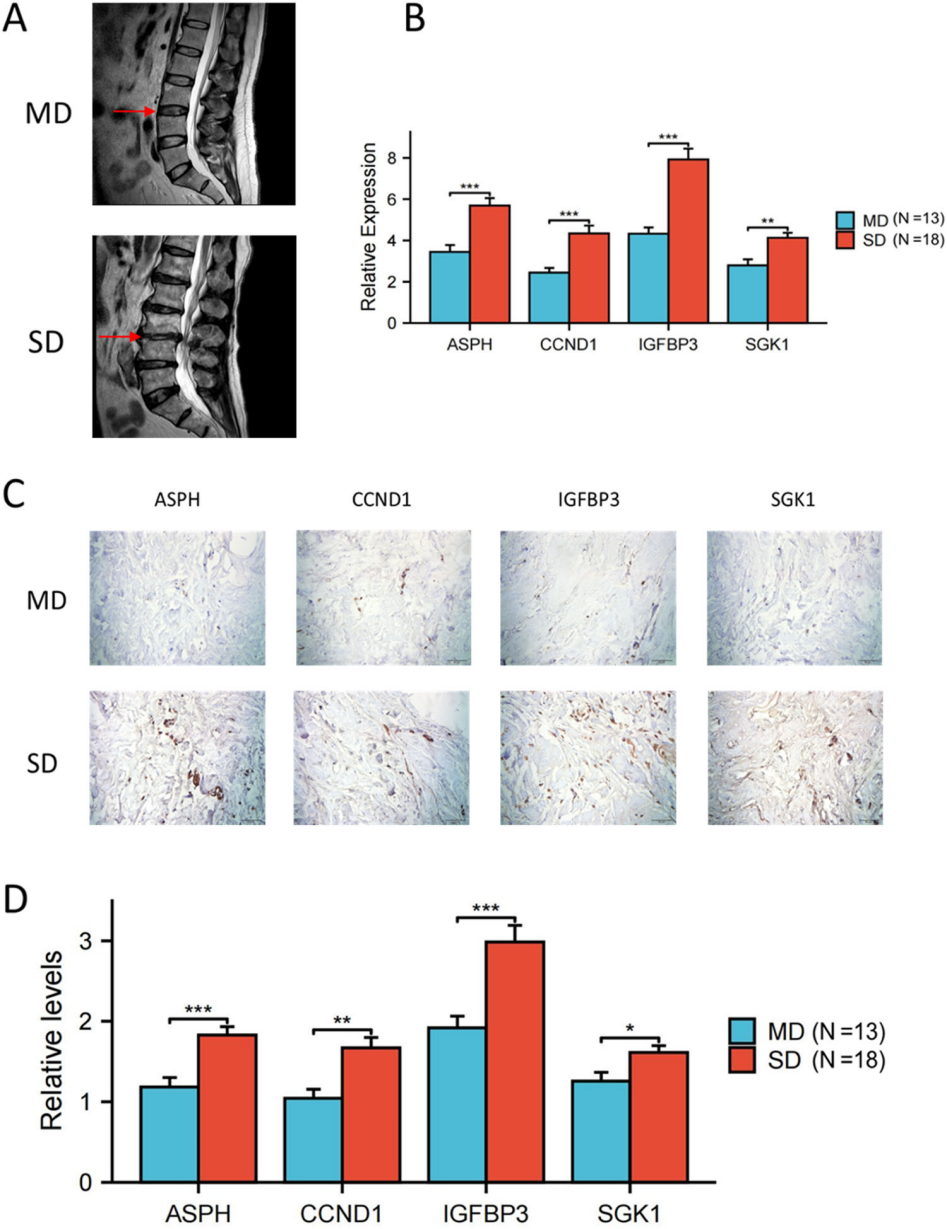
RT‐PCR and Immunohistochemical analysis of four hub SAGs in NP tissues from IDD patients. (A) MRI images showed lumbar disc herniation in MD and SD patients. (B) RT‐PCR analysis showed the relative levels of ASPH, CCND1, IGFBP3 and SGK1 in SD and MD group. (C) Representative immunohistochemical staining of disc tissues showed the expression of ASPH, CCND1, IGFBP3 and SGK1 in SD and MD group. (D) Statistical analysis for immunohistochemical staining results. *p < 0.05; **p < 0.01; ***p < 0.001.

The 31 samples (MD: 13 cases, SD: 18 cases) were collected for four hub SAGs expression validation. The representative magnetic resonance imaging (MRI) images of MD and SD groups were showed in Figure 12A. We then performed PCR analysis of the four hub SAGs in NP tissues from IDD patients. The relative levels of ASPH, CCND1, IGFBP3 and SGK1 were higher in the SD group compared with the MD group (Figure 12B). Immunohistochemical analysis obtained the similar results of PCR analysis (Figure 12C,D).

## 4 Discussion

IDD is considered to be the main cause for low back pain, which seriously affects people's normal work and quality of life. The pathophysiology of IDD is intricately linked to numerous factors, such as oxidative stress, cellular apoptosis, matrix metalloproteinases, and inflammatory mediators [36, 37]. The phenomenon of cellular senescence has garnered significant interest in research on age‐related diseases. Studies have indicated a strong correlation between IDD and cellular senescence in humans, with particular emphasis on the potential impact of NP cells senescence. However, the molecular mechanism of senescence‐related genes in IDD has only rarely been reported. In this research, we first identified hub SAGs and systematically investigated their roles in IDD using bioinformatics analysis.

We first screened four hub SAGs (ASPH, CCND1, IGFBP3 and SGK1) and validated in GSE23130 and GSE70362. ASPH is a type II membrane protein, and can regulate senescence and osteogenesis of bone marrow mesenchymal stem cells [38]. CCND1 is recognized as a regulator for the transition from G‐to‐S phase of the cell cycle. CCND1 deregulation results in abnormal proliferation, differentiation, and apoptosis of cells [39, 40]. It was reported that CCND1 and SNHG16 were upregulated in the cartilage tissues of osteoarthritis, and SNHG16 could promote the progress of osteoarthritis by regulating miR‐93‐5p/CCND1 axis. Yan et al [41] and Li et al [42] both identified CCND1 as a key gene for IDD through PPI network. IGFBP3 deacetylates multiple nonhistone proteins associated with apoptosis, which play important roles in cell proliferation, differentiation, aging and apoptosis [43]. SGK1 is an acute transcriptional regulator of multiple stimuli and influences the process of cell proliferation, apoptosis and inflammation [44]. A study found that SGK1 was upregulated in chondrocytes of osteoarthritis, and that SGK1 knockdown reduced catabolic and anabolic imbalances in chondrocytes [45]. In Parkinson disease, SGK1 was found overexpressed and SGK1 knockdown suppressed pro‐inflammatory activities of glia through inhibiting the NLRP3‐ and NFκB‐mediated inflammatory pathways [46]. These findings revealed the vital roles of SAGs in the development of various degenerative diseases. Our research demonstrated that the hub SAGs were significantly associated with multiple biological processes and pathways in IDD, such as angiogenesis, apoptosis, epithelial mesenchymal transition, inflammatory response, IL6‐JAK‐STAT3 and TGF‐β signaling pathway. Therapies targeting the hub SAGs and related pathways might become a promising treatment strategy for IDD patients.

The senescence process is characterized by alterations in the immune microenvironment, including the senescence of immune cells [47]. Research has identified immune dysregulation in IDD, such as aberrant macrophage polarization, dysregulated B cell expression, and abnormal T cell differentiation resulting in increased expression of inflammatory molecules [18]. These immune dysregulations have been significantly linked to the progression of IDD. It was reported that macrophages in degenerative intervertebral disc could secrete various inflammatory cytokines (TNF‐α, IL‐1 and IL‐6) to promote the development of IDD [48]. Takada found that the coculture of NP cells and macrophages could promote the release of IL‐8, IL‐6, COX‐2 and TNF‐α [49]. Type 1 helper (Th1) cells and type 2 helper (Th2) cells were reported to increase in intervertebral disc herniation. The infiltrated T cells were primed into IL‐4‐producing CD4 + Th2 cells and secreted various inflammatory cytokines, which contribute to the development of IDD and occurrence of pain [11]. While both senescence and immune dysfunction are present in the pathogenesis and development of IDD, the precise nature of their relationship remains uncertain. To solve this issue, we performed immune infiltration analysis, and found that SAGs were positively correlated with multiple immune cell types. Our results demonstrated that SAGs might promote the progress of IDD by regulating macrophages and T helper cells.

Nomogram is a prediction tool, designed to forecast disease occurrence and treatment outcome based on statistical modeling. Wu et al established a nomogram based on immune‐related genes for the diagnosis of IDD [50]. In this research, we developed a nomogram based on SAGs (ASPH, CCND1 and IGFBP3) to predict the risk of SD for IDD patients. The calibration curve and ROC curve both displayed the good performance of our nomogram. Although our nomogram was internally validated utilizing calibration curve, further research is necessary to externally validate the nomogram.

To accurately investigate the expression pattern of the four SAGs in intervertebral disc, we performed single cell analysis. Previous study illustrated that intervertebral disc cells could be classified into 4 cell types, including chondrocytes (nucleus pulposus cells) and fibroblasts (annulus fibrosus cells), epithelial cells and adipocytes [51]. Our study showed that degenerative intervertebral disc cells could be divided into five cell types based on their marker genes. Then we displayed the describe the expression pattern of the four SAGs: ASPH and SGK1 expressed in almost all cell types; IGFBP3 mainly expressed in epithelial cells; CCND1 mainly expressed in chondrocytes cluster 4 (the “repair cells” in nucleus pulposus). These results provide new understandings of SAG's roles in IDD at the cellular level.

Moreover, we used CDT database to predict potential drugs targeting SAGs, which have therapeutical effect for IDD patients. 11 drugs were finally identified: aflatoxin B1, benzopyrene, bisphenol S, dexamethasone, estradiol, indomethacin, progesterone, tetradioxin, tretinoin, trichostatin A and valproic acid. The therapeutic effect of several drugs for IDD or other degenerative diseases have been assessed by experiments. Dexamethasone is an effective anti‐inflammatory drug, which was reported to induce viability and chondrogenesis of IDD cells [52]. Estradiol was found to alleviate the degree of IDD by regulating the antioxidant enzyme and suppressing autophagy in rat model [53]. As a nonsteroidal anti‐inflammatory drug, indomethacin is the commonly used treatment for low back pain caused by IDD. A recent study showed that short‐term usage of indomethacin did not contribute to acceleration of IDD in rabbit model, and the long‐term effects of indomethacin for IDD remain unknown [54]. The protective effects of progesterone have been on revealed in the treatment of hereditary retinal degeneration and spontaneous motoneuron degeneration in animal experiment [55, 56]. Trichostatin A was reported to suppress synovial inflammation and subsequent cartilage destruction in arthritis mouse model [57]. However, drug‐related toxic effects may limit the studies on some of our predictive drugs. Future research including in vitro and in vivo experiments is necessary to ascertain the exact effects of our predictive drugs.

This study investigated the roles of SAG in IDD, identified four key SAGs, explored potential mechanisms, and ultimately predicted small molecule drugs targeting SAG. Nevertheless, the study has certain limitations. The data utilized for analysis were obtained from online databases, and the conclusions are predominantly derived from bioinformatics analyses. In future research, we will validate the impact of hub SAGs on NP cells through measurements of ROS levels, detection of SA‐β‐gal positive cells, and assessment of senescence‐related markers. Subsequently, we will investigate the potential of the predicted pharmacological agents identified in this study to counteract the effects of key SAG overexpression. Finally, IDD rat model will be established, and the intervertebral disc height and moisture content will be assessed using MRI and X‐ray imaging to further evaluate the impact of SAG on IDD in vivo.

## 5 Conclusions

Our study identified four hub SAGs (ASPH, CCND1, IGFBP3 and SGK1) correlated with IDD. Further analysis revealed that the 4 SAGs might regulate and multiple pathways immune infiltrations in IDD. The risk model based on the 4 SAGs showed good performance in predicting the risk of SD for IDD patients. At last, we predicted 11 drugs targeting the 4 SAGs which might have potential therapeutic effects for IDD patients. However, the data used for analysis was downloaded from online website, and the conclusion was mainly based on bioinformatics analysis. It is necessary to conduct in vivo and in vitro experiments to explore the functions of SAGs and the exact effects of our predictive drugs in IDD.

## Author Contributions

**Zijun Zhao:** conceptualization, data curation, formal analysis, resources, software, writing–original draft. **Yining Wang:** formal analysis, investigation, software, validation, writing–original draft. **Zairan Wang:** investigation, methodology, software, validation. **Fan Zhang:** methodology, validation, visualization. **Ze Ding:** validation, visualization. **Tao Fan:** conceptualization, methodology, project administration, supervision, writing–review and editing.

## Ethics Statement

Ethical approval for this study was obtained from the Ethics Committee of Sanbo Brain Hospital (SBNK‐YJYS‐2024‐030‐01).

## Conflicts of Interest

The authors declare no conflicts of interest.

## Supporting information

Supporting information.

Supporting information.

Supporting information.

Supporting information.

## Data Availability

The data set is available from GEO database.
