# Supplementary material for: Senescence in Intervertebral Disc Degeneration: A Comprehensive Analysis Based on Bioinformatic Strategies
Source: Immun Inflamm Dis. 2024 Nov 18;12(11):e70072. doi: 10.1002/iid3.70072 (PMC11571097; doi:10.1002/iid3.70072)
Supplement: Supplementary file 1 — Supporting information. [file IID3-12-e70072-s003.docx]

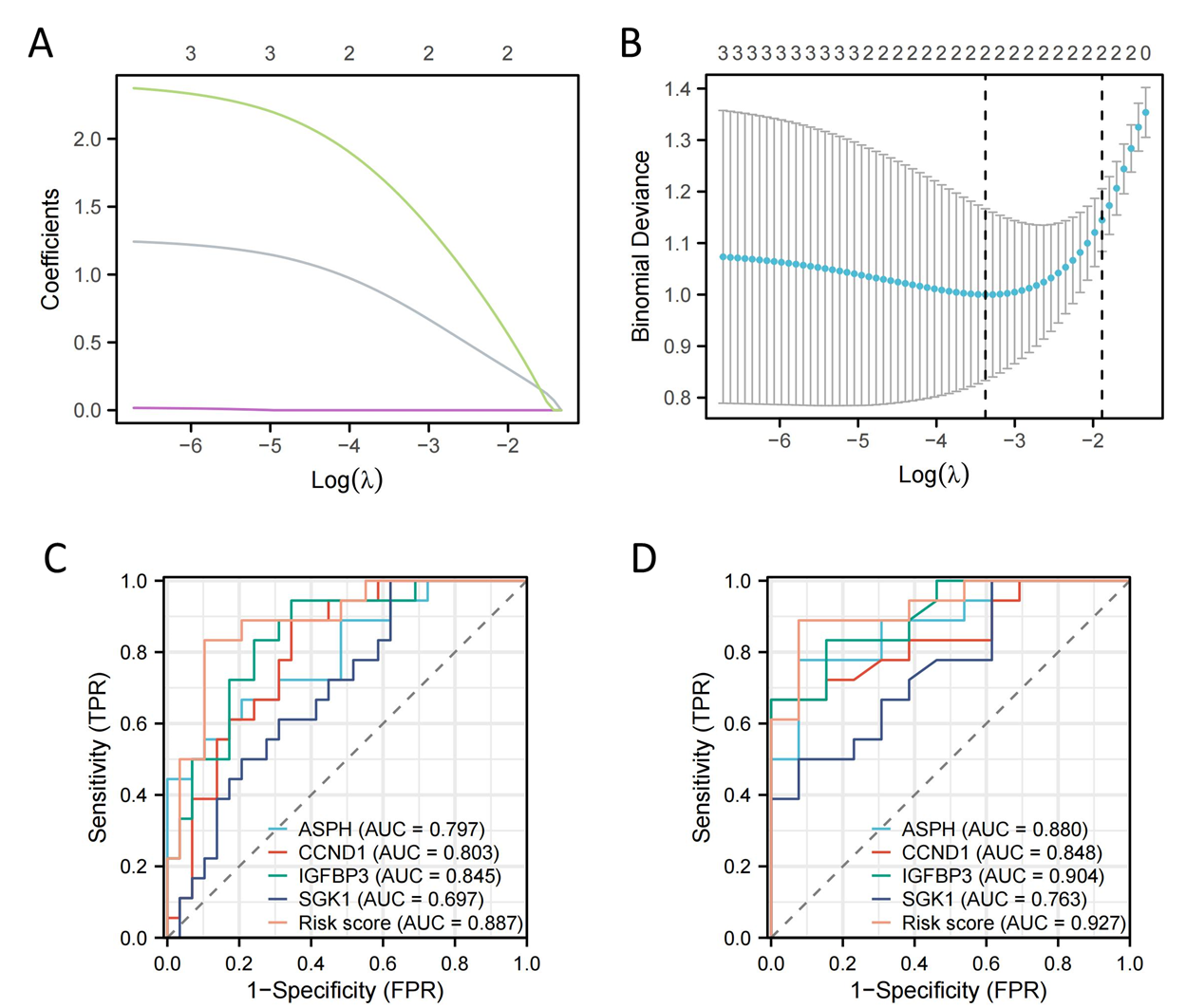


Figure S1. Establishment and validation of a LASSO model. (A) LASSO coefficient profiles of a model featuring the selected 2 genes. (B) Plots of 10-fold cross-validation error rates. (C) ROC curves for the 4 hub SAGs and LASSO model in training dataset. (D) ROC curves for the 4 hub SAGs and LASSO model in validation dataset.
